# Universal Health Coverage in Fragile and Humanitarian Contexts

**DOI:** 10.15171/ijhpm.2019.112

**Published:** 2019-11-23

**Authors:** Dick Chamla, Claudia Vivas-Torrealba

**Affiliations:** ^1^Health Section, Emergency Response Team, UNICEF, Three United Nations Plaza, New York City, NY, USA.; ^2^Health Section, UNICEF, New York City, NY, USA.

## Dear Editor,

Universal health coverage (UHC), a vision guiding current global health architecture and a Sustainable Development Goal that member states committed to achieve by 2030^1^ is possible if adequate resources and optimal financial and service delivery models reach over 30% of global populations living in fragile and humanitarian contexts. In 2017, fifteen countries, including 11 African countries, were classified as extremely fragile with most having ongoing humanitarian operations, five were classified as middle-income economies – Iraq, Nigeria, Syria, Sudan, and Yemen.^2^ In addition, 68.5 million people were forcibly displaced, 25.4 million of them hosted predominantly in Africa and the Middle East^3^ – regions with the most fragile and humanitarian situations.

Many will argue that fragility and humanitarian crises are among the strongest barriers to UHC beyond inaccessibility and unaffordability of health services. Invisibility of affected populations by lack of birth registration, and migration, often excluded from health access, is an equity issue at the heart of UHC. Conflicts and natural disasters, directly affect eight Sustainable Development Goal targets including UHC. High death toll recorded in these settings continue to face political backlash.^4^ Protection and security of health workers, a core commitment of international humanitarian law, are increasingly violated – further impacting access to quality health services. Yet, stable countries also face different challenges towards achieving UHC, ranging from climate change caused by industrial pollutions to the growing social inequalities, that leave behind a large segment of populations that cannot afford healthcare costs.^5,6^

## A Case for Health Reforms

Scientists have challenged traditional humanitarian service delivery as broken and in need of reforms.^7^ However, there is no international consensus on the best way of reaching UHC^1^ especially in constantly evolving fragile and humanitarian contexts. Evidence on sustainable financing in these contexts remains scarce despite high out-of-pocket payments as a percentage of health expenditure – from 78% in Afghanistan to over 81% in Yemen.^8^ Studies on effectiveness of pay-for-performance showed mixed results, with no significant increase in coverage of key interventions in countries like Afghanistan and Zimbabwe, but with positive results on quality of services.^9,10^ In Haiti, improvements in primary healthcare following pay-for-performance implementation, were boosted by international support, though unlikely to be sustained. Similarly, domestic public financing, the predominant source of health spending, continued to fall in countries that are fragile or in humanitarian crises.^11^ Health financing is equally affected in middle-income economies normally ineligible for donor funding. In most humanitarian response plans, and despite their rise, non-communicable diseases remain low on the priority ladder, while re-emergence of vaccine-preventable diseases such as diphtheria, and cholera outbreaks have become explosive in Yemen.^12^ Yet, many transformative propositions and opportunities such as UHC2030 convened by the World Bank, and Astana Declaration,^13^ to achieve UHC have remained largely upstream and yet to be optimized to achieve UHC in humanitarian settings.

## The Transformational Agenda

We propose a transformative agenda as a pathway to UHC in fragile and humanitarian contexts. First, sustainable humanitarian health financing through insurance systems funded by international support and government’s progressive taxation. Repackaging global instruments such as the Global Financing Facility, Global Fund, GAVI, the President’s Emergency Plan for AIDS Relief, and Cash Transfer programs could provide sustainable investments to the proposed insurance scheme. Second, new partnerships embracing the private-for-profit sector to activate health market approaches. Private sector engagement in Humanitarian Needs Overviews, and humanitarian response plans, will define roles and investments for private sector contribution, though few such partnerships exist. Third, ‘system thinking’ in health service delivery, recognizing that achieving UHC depends on complex interactions of interdependent partnerships, and interventions within and outside health sectors including participation of affected communities. What remains is defining the context-specific, multi-sectoral UHC package, building on the humanitarian-development nexus. Literature on the role of communities^14^ and other sectors eg, nutrition and child protection in improving health outcomes in humanitarian settings is expanding. This contrasts with current models of service delivery that are vertical and based on linear relationships between intervention and health outcomes, departing from current programming according to emergency phases – *preparedness, response, and early recovery/rehabilitation* . Fourth, is a need for new types of data and evidence, particularly in acute emergencies. For decades, the scale and severity of humanitarian emergencies have been defined based on crude and under-five mortality rates^15^ that are increasingly political, instead, data on *lives saved* , using robust modeling methodologies, could be less controversial.

In conclusion, UHC will remain a dream unless two billion people in fragile and humanitarian contexts are reached. Despite multiple global efforts, evidence on best financial and service delivery models to achieve UHC are missing, and urgently needed. A transformative agenda is proposed to ensure communities and health systems not only bounce back after shocks, but are transformed to achieve UHC.

## Ethical issues

Not applicable.

## Competing interests

Authors declare that they have no competing interests.

## Authors’ contributions

DC conceptualized and developed the first draft of this manuscript; CV reviewed and added health systems component of the manuscript. Both DC and VC conducted research search to inform the manuscript.

## Disclaimer

The views expressed in this article belong to the authors and not a reflection, direction or a policy of UNICEF.

## Authors’ affiliations

^1^Health Section, Emergency Response Team, UNICEF, Three United Nations Plaza, New York City, NY, USA. ^2^Health Section, UNICEF, New York City, NY, USA.
